# Biocompatibility of SU-8 and Its Biomedical Device Applications

**DOI:** 10.3390/mi12070794

**Published:** 2021-07-04

**Authors:** Ziyu Chen, Jeong-Bong Lee

**Affiliations:** Department of Electrical and Computer Engineering, The University of Texas at Dallas, Richardson, TX 75080, USA; z.chen@utdallas.edu

**Keywords:** SU-8, biocompatibility, biosensing, biomedical, implantable

## Abstract

SU-8 is an epoxy-based, negative-tone photoresist that has been extensively utilized to fabricate myriads of devices including biomedical devices in the recent years. This paper first reviews the biocompatibility of SU-8 for in vitro and in vivo applications. Surface modification techniques as well as various biomedical applications based on SU-8 are also discussed. Although SU-8 might not be completely biocompatible, existing surface modification techniques, such as O_2_ plasma treatment or grafting of biocompatible polymers, might be sufficient to minimize biofouling caused by SU-8. As a result, a great deal of effort has been directed to the development of SU-8-based functional devices for biomedical applications. This review includes biomedical applications such as platforms for cell culture and cell encapsulation, immunosensing, neural probes, and implantable pressure sensors. Proper treatments of SU-8 and slight modification of surfaces have enabled the SU-8 as one of the unique choices of materials in the fabrication of biomedical devices. Due to the versatility of SU-8 and comparative advantages in terms of improved Young’s modulus and yield strength, we believe that SU-8-based biomedical devices would gain wider proliferation among the biomedical community in the future.

## 1. Introduction

SU-8 is an epoxy-based negative-tone photoresist consisting of EPON SU-8 resin, solvent and a photoacid generator. Ever since its first introduction by IBM in the late 1980s, SU-8 has gained significant popularity in fabricating a wide range of devices including microelectromechanical systems (MEMS) devices. The primary reason for this popularity lies in the versatility of properties of SU-8. For example, SU-8 offers compatibility to conventional micromachining techniques such as spin-coating and photolithography to create well-defined features ranging from sub-micrometers to micrometers. Moreover, SU-8 can be coated to achieve films with thickness greater than 500 µm with a single layer and over 2 mm with multiple layers [1,2], which is very rare in microfabrication. Along with its unique very thick film, SU-8 is also known for its high resolution, and microstructures with height-to-width aspect ratios up to 100 have been demonstrated [3,4]. These capabilities have rendered SU-8 one of the ideal photoresists in microfluidic applications, such as microfluidic channels [5] and master molds for polydimethylsiloxane (PDMS) [6]. SU-8 is highly transparent in wavelengths greater than 400 nm, and exhibits large refractive index as well as low loss, which has allowed them to be a great material for optical waveguide application [7] as well.

In recent years, the emergence of biomedical MEMS applications has further advanced the utilization of SU-8 into innovative biomedical applications including wearable and implantable devices. Compared to conventional silicon, polymeric SU-8 offers excellent mechanical properties in terms of relatively low Young’s modulus (2~3 GPa) [8] and high yield strength. This has allowed SU-8 to be flexible but good to be utilized as a structural or functional component. With proper treatments, implantable MEMS devices, such as physiological pressure sensor [9], cantilever [10], neural probe [11], among others have been demonstrated. SU-8 surfaces can also be tailored via surface modification techniques to accommodate specific biomedical applications, such as the immobilization of biomolecules for biosensing [12], or the reduction in nonspecific adsorption of proteins for improved biocompatibility [13].

This review paper focuses on the biocompatibility of SU-8 and SU-8-based biomedical device applications. Its biocompatibility, surface modification techniques as well as various in vitro and in vivo applications are discussed, including platforms for cell culture and cell encapsulation, immunosensing, neural probes, and implantable pressure sensors. So far, comprehensive reviews on SU-8 biocompatibility and its surface modification techniques are scarce. It is our hope that this review paper would help elucidate the progress on the study of SU-8 biocompatibility and surface modifications to compensate the increasing interests of fabricating functional biomedical devices using SU-8.

## 2. Biocompatibility

Biocompatibility is often used to define the ability of a certain material to interface with biological tissues without inducing severe harm to the body in a specific application [14,15]. The biocompatibility of SU-8 has been extensively tested to evaluate SU-8 as a structural or functional material for wide range of biomedical applications. Depending on the specific application, the biocompatibility of SU-8 can be evaluated in vitro and/or in vivo. In vitro studies often involve the detection of leachates, toxicity to cells (also called cytotoxicity), cell attachment, and cell culturing. On the other hand, in vivo studies typically include implantation of the device and inspection of tissues at the site of use after a prolonged period. Although in vitro studies are easier to perform and potentially provide more quantitative results to evaluate biocompatibility, in vivo studies are more relevant. For example, neural devices are often implanted in the nervous system surrounded by delicate tissue and cells. Mismatch in the mechanical properties such as weight, shape and flexibility can cause severe adverse effects, such as cell and tissue damages, as well as inflammatory responses in the nervous system [16]. As a result, in vivo studies are often carried out in implantable devices to capture macroscopic systemic responses of host tissues.

### 2.1. In Vitro Studies

In vitro studies of SU-8 biocompatibility have been reported extensively. Interestingly, there are two contradictory conclusions about the biocompatibility of bare SU-8: one conclusion says bare SU-8 is not biocompatible and the other conclusion says it is biocompatible.

Several in vitro studies have reported the adverse effects caused by SU-8. Vernekar et al. reported that untreated SU-8 2000 is not cytocompatible to primary cortical or hippocampal neuronal cultures with only less than 10% of primary neurons surviving [17]. Assessment using cortical neuronal cell cultures showed that cortical neuronal cell viability next to SU-8 samples was significantly lower than control groups (plain polystyrene) at 21 days in vitro. Marelli et al. also reported that SU-8 does not support cell growth and adhesion of PC12 cell lines [18]. It was found that cell adhesion to pure SU-8 substrate is scarce compared to gold plated SU-8. Weisenberg et al. studied the hemocompatibility of SU-8 along with other common MEMS materials (i.e., silicon, silicon nitride, silicon dioxide, etc.) using human platelets [19]. Their results showed that platelet adhesion on SU-8 surface was significantly higher on SU-8, silicon, and silicon nitride surfaces, suggesting enhanced adhesion compared to control groups of polyurethane, parylene, and silicon dioxide. Since enhanced platelet adhesion is commonly used as a measure of thrombogenicity, it was suggested that SU-8 surfaces may be more reactive to human platelets and more thrombogenic. It has been postulated that the cytotoxic source of SU-8 might come from antimony (Sb) salt (i.e., triarylsulfonium hexafluoroantimonate) existing in the photoacid generator of SU-8 [20]. However, numerous subsequent studies have suggested that antimony leaching of cross-linked SU-8 may be small. X-ray photoelectron spectroscopy (XPS) and energy-dispersive X-ray spectroscopy (EDX) have been utilized to study the surface chemistry of SU-8. Ereifej et al. reported the presence of antimony on SU-8 using EDX. However, further analysis using XPS did not detect antimony, suggesting that the antimony presence on SU-8 surface is below the detection limit of 1% [21]. Walther et al. also confirmed the antimony on untreated SU-8 surface to be as small as 0.2 atm% [22].

In vitro studies in favor of SU-8 biocompatibility have also been extensively reported. Kotzar et al. first evaluated the cytotoxicity of SU-8 among other MEMS materials per ISO 10993-5 standards [23]. Their results showed that SU-8 can be classified as a low cytotoxic material with less than Grade 2 reactivity. Ereifej et al. reported in vitro test results utilizing C6 rat astrocytoma cell cultures, also confirming the cytocompatibility of SU-8. It is shown that cell viability on the SU-8 surface was at least 93% for up to 1 day in vitro with a higher initial cell attachment rate compared to control surfaces (silicon, platinum, or polymethyl methacrylate (PMMA)). These results have led the authors to conclude that SU-8 is a cytocompatible material. Numerous other studies utilizing different cell models such as SH-SY5Y human neuroblastoma cells [24] and primary cortical neurons [11] also favor SU-8 to support cell growth.

In a deeper study on identifying the potential cause of cytotoxicity, Nemani et al. studied the leaching of antimony from SU-8 in various solvents and buffers, such as phosphate-buffered saline (PBS), isopropanol, vegetable oil and phosphate buffers at different pH, and the antimony leachate was quantitatively evaluated using inductively coupled plasma mass spectrometry (ICP-MS) [25]. Their results showed that room temperature isopropanol sample with pH 5.5 exhibited maximum leaching of Sb of 23.4 ppb, while hydrophobic vegetable oil and hydrophilic PBS demonstrated reduced leaching. It is suggested that the enhanced Sb leaching observed at acidic pH might be a result of SU-8 etching in an acidic environment. Further quantitative analysis of the cytotoxicity of Sb leachates was performed by using MTT (3-(4,5-dimethylthiazol-2-yl)-2,5-diphenyltetrazolium bromide) assay on 9L glioma cell line. The results showed that SU-8 extracts with PBS did not inhibit viable cell growth for 2.5% and 5% extracts, while 10% extract exhibits a significant inhibition of cell growth. Hemolytic activities of SU-8 sample were also found to be of a comparable level to three Food and Drug Administration (FDA)-approved biocompatible control groups, including silicon elastomer (SE), Buna N, and medical steel (MS).

The apparent inconsistent conclusions about the biocompatibility of bare SU-8 have been observed by many other researchers. It has been suggested that the biocompatibility of SU-8 may be influenced by cell lines, photoresist formula, and fabrication variances, such as ultraviolet (UV) exposure and baking time [25,26]. Therefore, it is advisable to test the biocompatibility of fabricated SU-8 structures with specific cell lines with specific fabrication recipes in interest in vitro before building SU-8-based biomedical devices.

**Figure 1:**
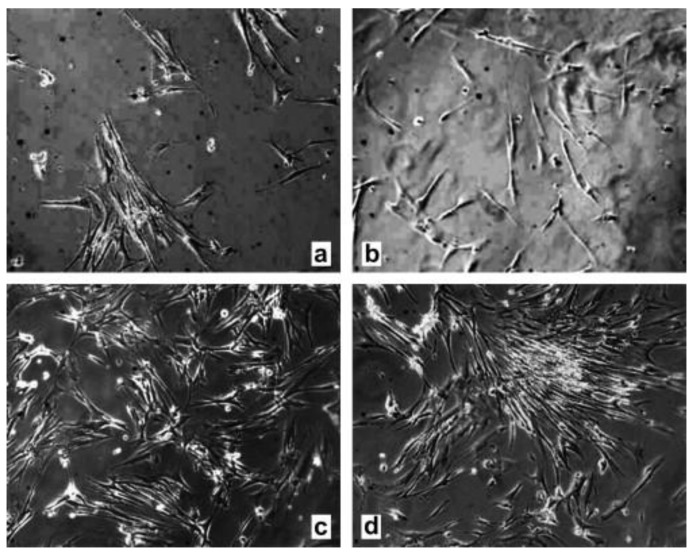
Representative images (10×) of MRC-5 cells after 3 days of cultivation on (**a**) plasma activated soda-lime glass, (**b**) an untreated SU-8 surface, (**c**) an oxygen plasma activated SU-8 with a dose of 2.77 J/cm^2^ and (**d**) 22.2 J/cm^2^. Reprinted from Hennemeyer et al. [27] with permission from Elsevier.

It is known that various surface treatments including heat treatment, isopropanol ultrasonication, O_2_ plasma treatment, and parylene coating, can be used to improve the biocompatibility of SU-8. Vernecker et al. evaluated the effectiveness of various surface treatments in terms of cell viability cultured on treated SU-8 surfaces [17]. Their results showed that 3-day heat treatment at 150 °C under vacuum improves the viability rate to 45.8% ± 4.5%, while combined treatments with 25 µm parylene coating, heat and isopropanol ultrasonication further enhance the viability of cells to 86.4% ± 1.9%. Hennemeyer et al. found that O_2_ plasma treatment greatly improve cell proliferation of SU-8 from 50 cells/mm^2^ to 350 cells/mm^2^ [27]. Figure 1 shows the cell proliferation of untreated and treated SU-8 for MRC-5 cells.

### 2.2. In Vivo Studies

In vivo studies of SU-8 based implants have also been reported extensively, which involve the implantation of SU-8 structure or device at the site of use. Although biocompatibility of implants is generally more complicated in nature affected by numerous factors (i.e., flexibility, shapes, material, etc.), in vivo assays are more relevant and able to capture macroscopic biological responses for implantable MEMS devices [16]. Kotzar et al. performed in vivo tests of SU-8 implants following ISO 10993-6 guidelines [23]. Rabbit model was used for SU-8 implantation with periods of 1 and 12 weeks, and subsequently the implantation sites were examined macroscopically to evaluate local tissue responses, such as signs of inflammation. Their test results led them to categorize SU-8 implant as non-irritant during short-term and long-term implantation of 1 and 12 weeks in rabbits. Voskerician et al. utilized SU-8 microstructures in cylindrical stainless steel wire mesh cages and implanted it subcutaneously in rats to study the in vivo biocompatibility of SU-8 implants [28]. Their results show that SU-8 is comparable in inducing inflammatory responses to other common MEMS materials including gold, silicon dioxide, etc. Nemani et al. carried out in vivo histocompatibility studies in accordance with ISO 10,993 recommendations and found a muted immune response to subcutaneous SU-8 implants in rats. Huang et al. reported that a SU-8 neural probe implanted in rat did not induce apparent astrocyte aggregation in the cortex and striatum regions.

**Figure 2:**
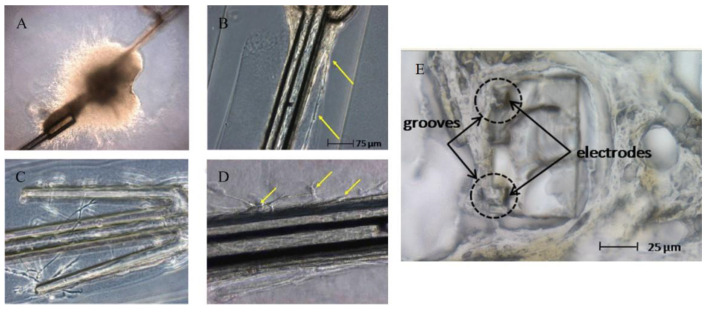
Optical images of: (**A**) explanted DRG from four-day old rat, cultured for 14 days on two separate SU-8 microprobes shown placed at 8 and 2 o’clock relative to the DRG explant; (**B**) neural fibers growing away from the DRG explants, adhering to every surface along the full length of the SU-8 microprobes which appear to be guiding the growth of fibers along its shaft (arrows); (**C**) its flexible wing extensions (calibration 75); (**D**) close up of neurons (arrows) migrating away from the DRG explants along the shaft of the SU-8 microprobe; and (**E**) cross-sectional view of the SU-8 microprobes recovered from regenerated peripheral nerves 17 weeks after tubulization showing that groove electrodes were filled with nerve tissue. Robust fiber spike signals (signal-to-noise ratio > 3) were recorded throughout this implantation period using these grooved electrodes © 2008 IEEE. Reprinted from Cho et al. [29] with permission.

However, these studies are mostly limited to SU-8 flat substrates which do not mimic intricate 3D microstructures found on many SU-8-based functional devices. While the results indicate the material biocompatibility of SU-8, biocompatibility tests with completely fabricated SU-8 devices are more relevant. Cho et al. reported the test results of the SU-8 microprobes with bipolar longitudinal gold electrodes in grooves for neural interface applications [29]. First, in vitro biocompatibility tests were performed by using three types of cell lines, including human skin fibroblast cells, Schwann cells for myelination of mature peripheral nerve fibers, and neurons from explanted dorsal root ganglia (DRG) for the growth of sensory fibers in peripheral nerves. Figure 2A–D shows the optical images of nerve cell cultures thrive at the site where the SU-8 microprobe was at direct contact with DRG explants, as well as nerve fibers which grew away from the explants along the SU-8 microprobes. Their results showed that SU-8 neural probes provide a biocompatible platform for the growth and migration of DRG cells and nerve fibers without any signs of cytotoxicity. The microprobes were implanted in rat model to test the functionality of the microprobes. Subsequent examination of the nerve tissues at the implantation sites showed no evidence of infection or inflammation. Figure 2E shows the cross-section of a recovered SU-8 microprobe 17 weeks after tubulization with nerve tissues covering the groove electrodes, and showed no signs indicating fibrous encapsulation caused by the implanted SU-8 microprobes.

Márton et al. conducted a quantitative study on the biocompatibility of SU-8-based neural probes implanted in neocortex regions for 2 months period using rat model [30]. Their results showed that neuron density decreased to 24 ± 28% with respect to controls at distance less than 20 µm from the implant, 74 ± 39% at 20 to 40 µm distance, and comparable level at distance greater than 40 µm. Examinations also revealed that the glial scar thickness was only 5 to 10 µm thick. The results suggest that the adverse effects induced by SU-8 neural probes are localized to a very small region around the implants.

## 3. Surface Modification

**Figure 3:**
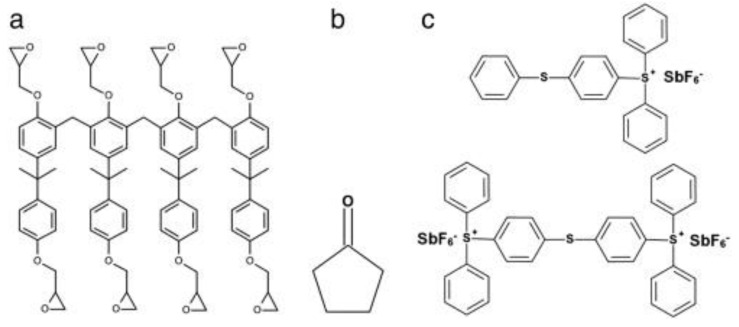
(**a**) Chemical composition of SU-8 photoresist. (**a**) SU-8 2075 comprises SU-8 monomer, (**b**) a solvent, cyclopentanone, and (**c**) a photoacid initiator, mixed triarylsulfonium hexafluoroantimonate salts. Reprinted from Nemani et al. [25] with permission from Elsevier.

Surface modifications have been reported to functionalize SU-8 for biomedical applications. As shown in Figure 3a, cross-linked SU-8 consists of eight epoxy rings on each monomer. Various dry and wet chemical treatments to SU-8 surfaces have been reported to open the epoxy rings to hydrophilize SU-8 or immobilize biomolecules.

SU-8 is generally considered as a hydrophobic material with static contact angle ranging from 74° to 90° [22]. The hydrophobicity has greatly limited SU-8 in biological applications due to increased nonspecific adsorption of biomolecules and reduced cell attachment. Oxygen plasma treatment [27] has been utilized as an effective method to render SU-8 surfaces hydrophilic with a water contact angle (WCA) less than 5°. The decrease in contact angle is attributed to the generation of functional groups such as carboxyl groups due to the opening of epoxy rings as well as increased surface roughness [31]. The increased number of functional groups has been utilized to enhance hydrophobicity for cell proliferation [27] and immobilize biomolecules [32]. Similar to plasma treatment, Ozone/UV treatment [33] has also been reported to render SU-8 surfaces hydrophilic with water contact angles less than 30°.

However, these treatments impose additional challenges as well. The hydrophilic behavior resulted from plasma treatment is temporary rather than permanent. The hydrophilic SU-8 recovers to hydrophobic within a time period ranging from days to months [22]. In addition, plasma treatment causes significant increase in antimony from 0.2 atm% to 2.6 atm% on the surface of SU-8, which might induce cytotoxicity [22,31]. However, the increase is most probably in the form of Sb_2_O_5_, where antimony exists as Sb (V) rather than Sb (III) [22]. Although the toxicity of Sb has been widely known, it has been reported that the Sb (V) compounds are less toxic than Sb (III) [34], suggesting less cytotoxicity due to O_2_ plasma treatment.

Physical adsorption or chemical modification have also been reported to modify SU-8 surfaces. Various biomolecules, such as collagen or gelatin, have been utilized to coat SU-8 surfaces for improved hydrophilicity and enhanced cell attachment and proliferation [35,36,37]. Chemical modifications on SU-8 surfaces have also been reported using sulfuric acid [37,38] or cerium (IV) ammonium nitrate (CAN) [39]. The water contact angles were found to decrease from an average of 103.8° to 45.1° on gelatin-coated SU-8 surfaces, and 81.7° on sulfuric acid treated surfaces [37]. These methods rely on residual epoxy rings on cross-linked SU-8 surfaces by converting them into hydroxyl groups to improve hydrophilicity. These modification methods, however, are limited in their ability to tailor surface properties of SU-8 for the specific adsorption of biomolecules and relatively low density of surface functional groups. Moreover, the use of sulfuric acid or CAN is undesirable as these wet chemicals are highly corrosive and impose significant health risks to human health. Stangegaard et al. also found that SU-8 surfaces treated with only HNO_3_-CAN induced different gene expression levels between HeLa cells grown on these treated SU-8 surfaces and control groups [40].

An alternative strategy to modify SU-8 is the grafting of functional groups or polymers to tailor SU-8 surfaces for enhanced cell attachment or biomolecule immobilization. Joshi et al. utilized hot wire chemical vapor deposition (HWCVD) to graft amine groups onto SU-8 surfaces for the immobilization of biomolecules [38]. Marie et al. reported the immobilization of deoxyribonucleic acid (DNA) to SU-8 by the condensation of amine groups with epoxy rings on the surface of SU-8 [41]. On the other hand, SU-8 surfaces have been modified by graft polymerization with a wide variety of monomers, including polyacrylic acid (PAA), polyethylene glycol (PEG) or its analogues. These polymers have been known to minimize nonspecific protein adsorption and thus reduces biofouling [15], as well as enhance wettability and cell attachment [42]. Various methods have been reported by using wet chemical CAN treatment [42], UV irradiation [20], or O_2_ plasma treatment [43,44] to convert residual epoxy rings on SU-8 surfaces into hydroxyl groups as initiation sites for subsequent grafting polymerization.

## 4. Applications

### 4.1. 3D Structures for In Vitro Studies

As a thick film photoresist, SU-8 is highly desirable in the fabrication of high-aspect-ratio structures with standard spin-coating, photolithography, and developing processes. This versality has allowed SU-8 to be utilized as a structural material to construct very thick 3D microstructures that are otherwise difficult to achieve. As a result, SU-8 has been widely used to fabricate mold masters for microfluidic organs-on-a-chip devices based on PDMS (polydimethylsiloxane) [45,46,47].

**Figure 4:**
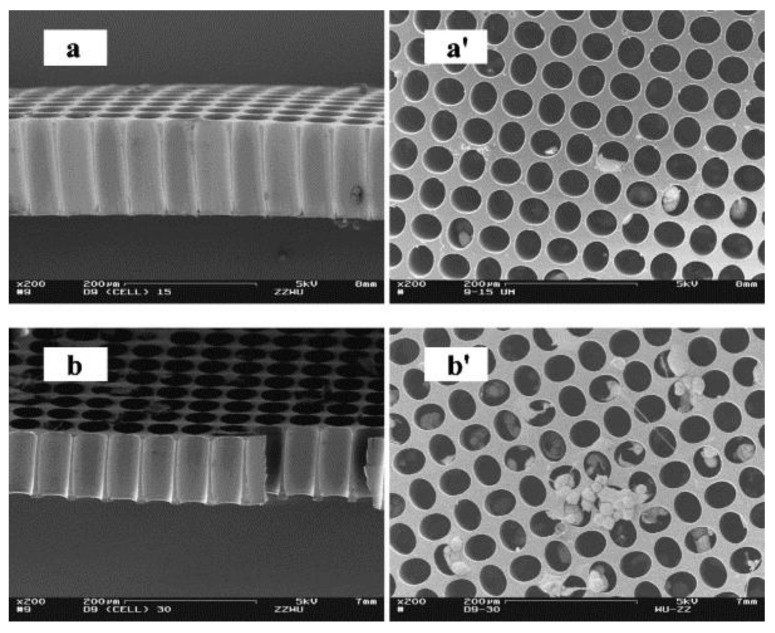
Scanning electron microscopy (SEM) images showing cross-sectional profiles of the 50-μm diameter microwell patterns (**a**,**b**) and SH-SY5Y cells cultured on day 4 into differentiation on the patterns (**a′**,**b′**). Pattern thicknesses were 146 μm (**a**,**a′**) and 97 μm (**b**,**b′**). Bar = 200 μm. Reprinted from Wu et al. [49] with permission from Elsevier.

Alternatively, SU-8 have been utilized directly as the functional materials for intricate 3D structures. One of such works demonstrated by Choi et al. utilized SU-8 in the fabrication of a 3D scaffold structure for neural cell culturing [48]. A series of high-aspect-ratio towers with tower diameters ranging from 20 to 200 µm and height of over 700 µm were fabricated using standard UV lithography processes. Wu et al. demonstrated the successful integration of SH-SY5Y human neuroblastoma cells to SU-8 microwell structure with diameter of 100 µm and an aspect ratio of approximately one [49] (Figure 4). Their results show that the cell density was higher on the shallower microwells (97 µm) than the deeper ones (146 µm). Moreover, neuronal extension, cell attachment to sidewalls, as well as cellular community formation inside of microwells were observed in the 97 µm wells. These results indicate that the microwell structure might be more suitable for promoting neural cell activities than flat substrates.

**Figure 5:**
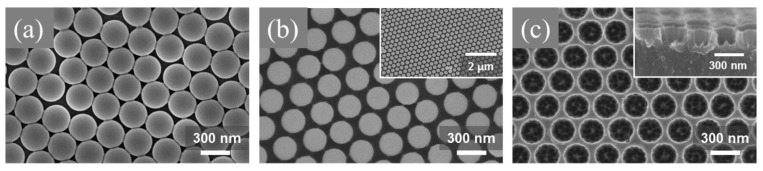
SEM images during nanoporous SU-8 substrate fabrication. (**a**) A monolayer of polystyrene nanoparticles on the SU-8 substrate, (**b**) etched polystyrene nanoparticles on the SU-8 substrate and the inset showing uniformly etched PS nanoparticles over the SU-8 substrate, and (**c**) fabricated nanopores on the surface of SU-8 and the inset showing the cross-sectional view of the nanoporous SU-8 substrate. Reprinted from Kim et al. [50] with permission from Elsevier.

SU-8 surfaces with nanopores have also been fabricated and investigated for enhanced cell attachment. Kim et al. utilized a combination of polystyrene (PS) nanoparticles and patterned chromium (Cr) layer as an etching mask to create nanopores in SU-8 [50]. Figure 5 shows the SEM images of the fabrication processes for the nanoporous SU-8 substrate. The fabricated nanopores were measured to be approximately 240 nm in diameter and 300 nm in pitch length. The nanoporous SU-8 surfaces were evaluated using rat pheochromocytoma (PC12) cell line, and it was found that these surfaces favor cell differentiation and cell migration compared to flat control samples.

**Figure 6:**
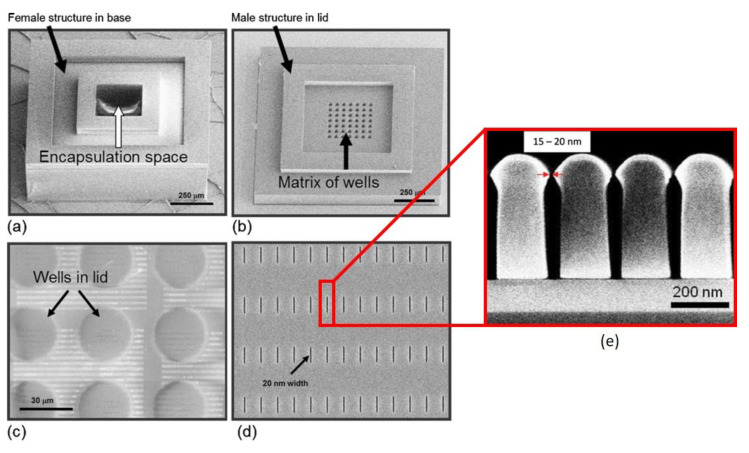
SEM images of the microcontainer’s hollowed cubic base (**a**), and lid that contains a matrix of wells that exposes a thin nanoporous membrane at the base of the wells (**b**). The thin nanoporous membrane has a nanopore array which is shown magnified in panel (**c**). The nanopores (enlarged view, **d**) permit selective molecular transport through the membrane. Reprinted from Gimi et al. [51] with permission from Springer. (**e**) Cross section of the nanotrenches in SU-8. The topmost nanoslot opening is as narrow as 15 nm. Reprinted with permission from Kwon et al. [52]. Copyright 2009, American Vacuum Society.

**Figure 7:**
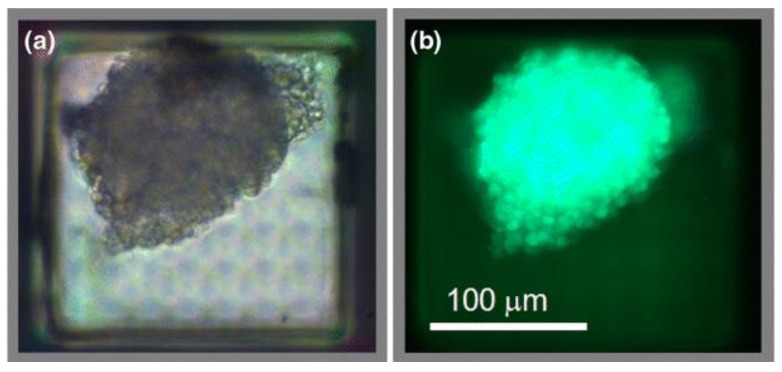
The transparent microcontainer was devised to facilitate optical imaging of the encapsulated cells. A representative phase contrast micrograph (**a**) and fluorescent image acquired in the green channel (**b**) of a cluster of ~3000 9L-3HRE-luc/GFP cells encapsulated within the sealed microcontainer. Reprinted from Gimi et al. [51] with permission of Springer.

Apart from enhancing cell attachment and growth, 3D structures can also be used to encapsulate cells capable of producing therapeutic biomolecules for potential treatment purposes. However, the encapsulation of such cells requires a nanoporous structure to isolate large immune proteins, yet allows the passage of nutrients (i.e., oxygen), waste, and therapeutic molecules. Gimi et al. and Kwon et al. first designed and reported a SU-8 microcontainer with nanopores within the surface of the container [51,52]. The fabrication strategy utilized electron beam lithography (EBL) to create well-defined nanoscale features and O_2_ plasma reactive ion etching (RIE) to create a dense array of nanopores with the opening of 15~20 nm in SU-8 membrane. Figure 6 shows the SEM images of the fabricated SU-8 microwell structure and lid with nanopores, as well as close-up view of the cross-section with 15~20 nm wide opening at the top of the nanopores. The fabricated device was tested in vitro using 9L rat glioma cells. Figure 7 shows the phase contrast micrograph and fluorescence image of ~3000 cells encapsulated in the microcontainer. Their results show that cells encapsulated in microcontainers without nanoporous membrane yielded markedly higher bioluminescence than those in nanoporous structures, which indicates improvement of the oxygenation of cells through the dense array of nanopores.

### 4.2. Biosensing

**Figure 8:**
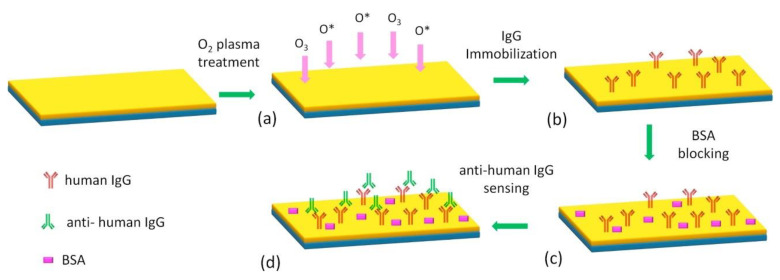
Schematic representation of the immobilization process of IgG on SU-8 substrate and of anti-human IgG binding: (**a**) oxygen plasma treatment for nanotexturing and surface chemical modification of SU-8, (**b**) IgG immobilization on surface, (**c**) blocking with a BSA-based solution, (**d**) incubation of anti-human IgG to evaluate the bioactivity of the immobilized IgG. Reprinted from Grimaldi et al. [53] with permission from Elsevier.

SU-8-based biosensors have been extensively reported in recent years thanks to their combined favorable optical, mechanical and chemical properties. For example, the residual epoxy rings on cross-linked SU-8 are often utilized to immobilize biomolecules like antigens or antibodies. The immobilization of biomolecules onto SU-8 surfaces often includes the opening of epoxy rings to form surface functional groups, such as hydroxyl or amino groups [31]. Afterwards, biomolecules are covalently bonded to these surface functional groups. Various treatments, including dry and wet chemical treatments have been reported to open residue epoxy rings on cross-linked SU-8. O_2_ plasma treatment similar to the hydrophilization of SU-8 has been utilized to immobilize human Immunoglobulin (IgG) antibody or similar biomolecules [44,53]. Figure 8 shows the schematic diagram of IgG immobilization through O_2_ plasma treatment. Alternatively, wet chemicals such as CAN (cerium(IV) ammonium nitrate) [54] or silanization treatment with APTMS (3-aminopropyltrimethoxysilane) and GTA (Glutaraldehyde) [32] can be used to create surface functional groups for the covalent binding of biomolecules onto SU-8. Additionally, it has been found that DNA can be directly coupled to SU-8 surfaces through the condensation of primary and secondary amine groups from DNA with the residual epoxy rings from SU-8 [41].

In addition to the immobilization of biomolecules, SU-8 are utilized as a functional or structural element in the construction of biosensors utilizing various detecting principles. Calleja et al. utilized a surface functionalized SU-8-based polymeric cantilever and optical readout to detect immunoreactions between the human growth hormone (hGH) and its antibody (α-hGH) [55]. The fabricated SU-8 cantilever was treated with sulfuric acid to open the epoxy rings of SU-8 for subsequent silanization and immobilization of hGH antigens. Compared to gold-coated silicon cantilevers, SU-8-based cantilever was found to exhibit higher tolerances to ambient environmental changes like temperature, pH or ion concentration fluctuations, and resulted in reduced noises and improved sensitivity.

**Figure 9:**
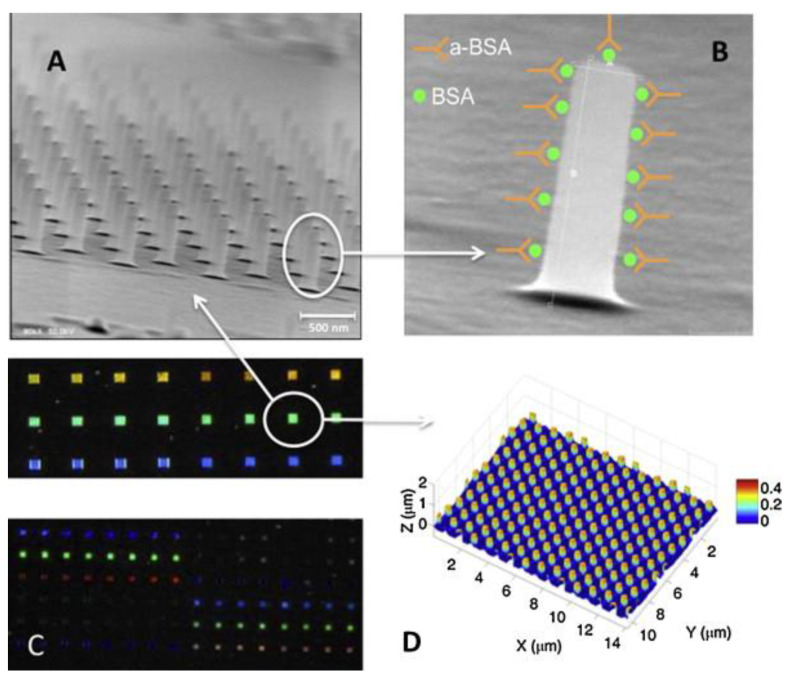
(**A**) SEM micrograph of a SU-8-based BICELL, (**B**) SEM micrograph of one single nano-pillar and a schematic representation of BSA immobilization and aBSA recognition, (**C**) Optical image of a biochip with a number of BICELLs, (**D**) Confocal image (Leica DCM3D) of one of the BICELLs after the infiltration experiments. Reprinted from Holgado et al. [56] with permission of Elsevier.

Other approaches utilizing photonic methods have been reported. Holgado et al. utilized a SU-8 nanopillar arrays-based biomolecule detector [56]. Figure 9 shows the SEM image of the SU-8-based photonic biosensor. The photonic device is sensitive to different concentrations of biomolecules, which results in change in optical responses of the reflective spectra. Their results showed that the device exhibits a maximum detection sensitivity of 2 ng/mL for bovine serum albumin (BSA) antigen and anti-BSA antibody (aBSA) immunorecognition. Alternatively, due to the high optical transparency of SU-8, it has also been used as waveguide materials in various applications including biosensors. Mach-Zehnder Interferometer (MZI) [57] and evanescent wave spectroscopy [7]-based SU-8 waveguide setup have also been reported to detect immunoreaction with a sensitivity of 1 ng/mL and 1.5 µg/mL, respectively.

### 4.3. Functional Devices for In Vivo Applications

As a polymeric material, SU-8 is promising as a structural material to construct implantable devices for biomedical applications due to its reduced Young’s modulus, improved yield strength and biocompatibility. A wide range of SU-8 functional devices have been fabricated and reported, including microneedles, neural probes, and sensors.

#### 4.3.1. SU-8 Based Microneedles

**Figure 10:**
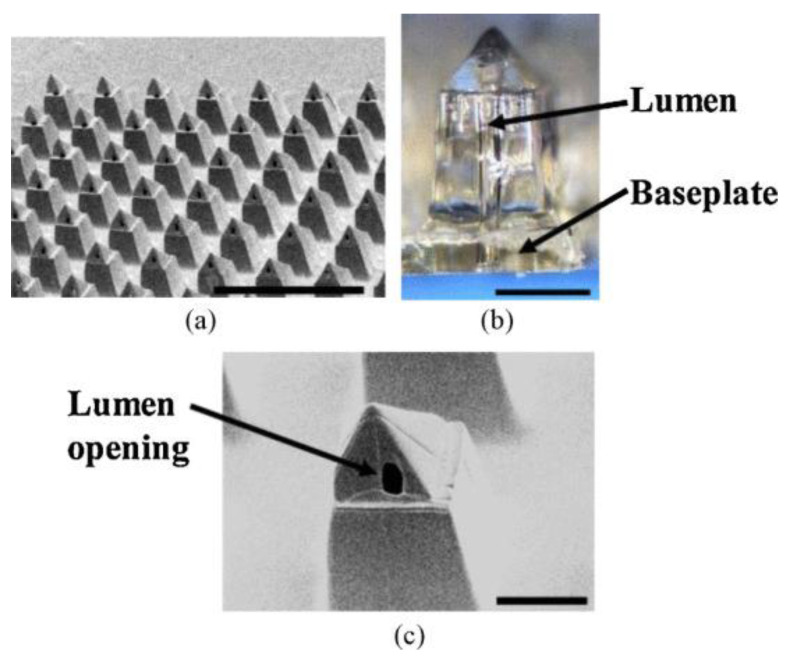
(**a**) An SEM image of bird’s-eye view of fabricated microneedle array coated by 15 nm Cr/150 nm Au for SEM imaging. (**b**) An optical micrograph showing a fabricated hollow microneedle with a baseplate. (**c**) An SEM image revealing the pyramidal tip with a lumen opening and upper shaft. The scale bar is 2 mm, 400 μm, and 250 μm in (**a**–**c**), respectively. © 2013 IEEE. Reprinted from Wang et al. [58] with permission.

As a promising replacement for traditional hypodermic needles, polymer-based microneedles have attracted significant attentions. Since SU-8 offers favorable properties such as sufficient mechanical strength, improved biocompatibility as well as ease of fabrication, it has been utilized to create microneedles for transdermal drug delivery. Wang et al. reported an SU-8 microneedle array with hollow pyramid structures using a combination of PDMS mold casting and UV lithography processes [58]. The fabricated SU-8 microneedle arrays are 825 μm in height and 400 μm in width (Figure 10). Mechanical characterizations using porcine skin showed that the insertion force and fracture force of a single needle are 2.4 N and 90 N, respectively.

**Figure 11:**
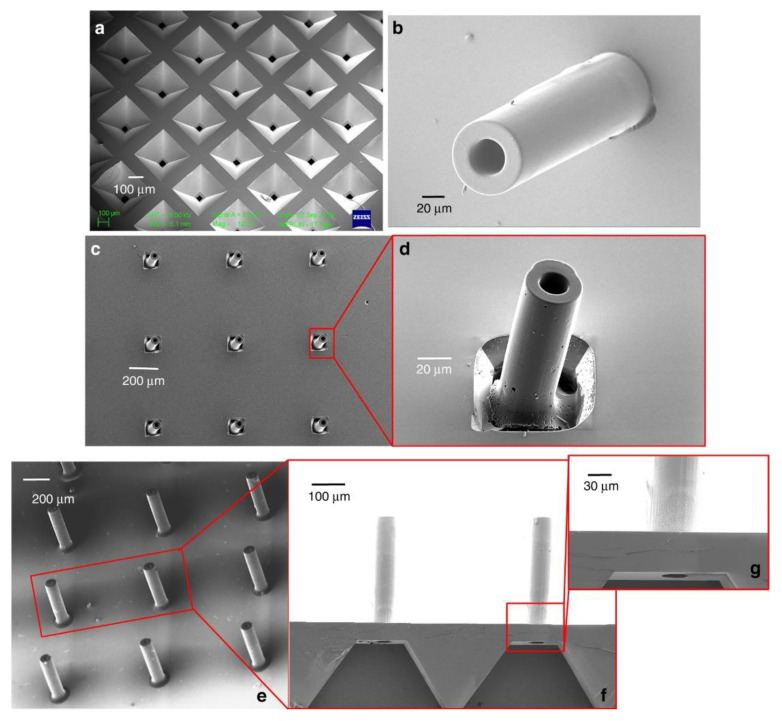
(**a**) Etched microfluidic conduit in silicon through which the drug flows from the drug reservoir to the CMN. (**b**) SMN fabricated on a microfluidic conduit backside of the image shown in (**a**). (**c**) CMN array formed after pyrolysis. (**d**) Magnified view of a CMN. (**e**) Optimized CMNs aligned on etched microfluidic ports on a silicon wafer. (**f**,**g**) Magnified images of the CMNs and the underlying flow channel. Reprinted from Mishra et al. [65] with permission from Springer Nature.

SU-8 has also been exploited to create high-aspect-ratio SU-8 microneedles. There have been numerous works on microneedles that utilized SU-8 either as a structural material [59,60,61] or a mold for creating hollow metallic microneedles [62,63,64]. Figure 11 shows the SEM image of one the fabricated SU-8 microneedle arrays using SU-8 itself as microneedle structural material. Mishra et al.’s work started with the fabrication of high-aspect-ratio SU-8 hollow structures [65]. The hollow SU-8 structures were then subsequently pyrolyzed in an inert atmosphere at 900 °C to convert the SU-8 structures into glassy carbon structures. The glassy carbon hollow microneedle showed higher Young’s modulus and hardness compared to SU-8 before pyrolysis. Chaudhri et al. demonstrated SU-8 microneedles with an inner diameter of 100 μm and a wall thickness of 15 μm, as well as a height of 1540 μm and an achieved height-to-width aspect ratio of ~103 [61].

#### 4.3.2. SU-8 Neural Probes

Neural implants have been a major research topic in the field of neuro prosthetics during past few decades. Implantable neural probes can be utilized to interface with neurons and electronics to record neural signals or to stimulate of neurons. These devices can potentially be used to deepen the understanding of cerebral functions as well as control external prosthetic limbs or robots by neural signals [66]. Silicon-based neural probes have been developed using standard microfabrication processes [67], however, it was found that these devices are quite limited in neuronal applications due to the rigid and brittle nature of silicon. These silicon probes are prone to break during operation, as well as increased risks of tissue inflammation or traumatic damage to brains [66]. Polymeric materials like SU-8 have been subsequently utilized as structural material for neural probes due to their improved flexibility, yield strength and reduced risks to tissue damages. Moreover, it was found that SU-8 can be used to substitute toxic chromium (Cr) as an adhesion layer for gold (Au) [68]. Their results showed that Au/SU-8 electrodes provided comparable adhesion yet improved biocompatibility, in contrast to a traditional Cr/Au electrode. These advantages have rendered SU-8 a superior material to be utilized in neural probes or microelectrode arrays (MEA).

**Figure 12:**
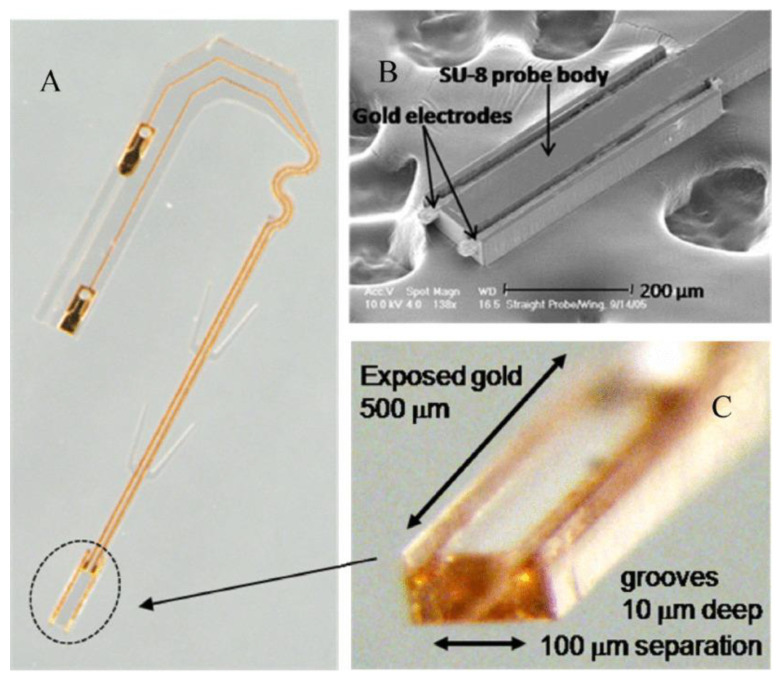
(**A**) Optical image of the “J-shaped” SU-8 microprobe featuring bipolar gold electrodes recessed in “grooves” designed to guide fiber growth, “wings” to maintain central position within implant channels, serpentine feature for greater flexion, and wire bonding pads. (**B**) SEM image of bipolar gold electrodes in recessed grooves. (**C**) Close-up image of bipolar groove electrode tip. © 2008 IEEE. Reprinted from Cho et al. [29] with permission.

Cho et al. reported SU-8 neural probes with a longitudinal electrodes in grooves design for neural spike signal recording [29]. Figure 12 shows the optical as well as SEM images of the fabricated SU-8 microprobe. The device was successfully implanted into the sciatic nerves of rats for evaluation of long-term in vivo biocompatibility. Their results showed that the 13 rats surgically implanted with the SU-8 neural probes exhibit no signs of tissue inflammation or damage reactions during an extended period ranging from 4 to 51 weeks with successful continuous neural spike detection during this extended period.

**Figure 13:**
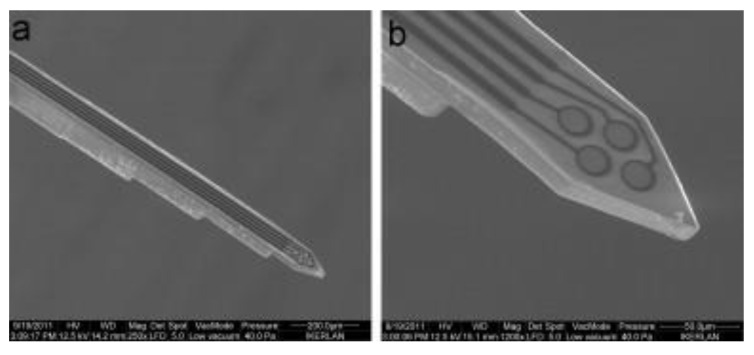
SEM pictures of: (**a**) a layered SU-8 probe and (**b**) the tetrode at probe surface level. Reprinted from Altuna et al. [71] with permission of Elsevier.

SU-8 as a versatile photoresist has allowed for innovative designs in neural probe devices. Marchoubeh et al. reported an SU-8-based neural probe for electrochemical analysis in neural regions [69]. The fabricated device was shown to detect potassium and dopamine in potassium chloride electrolyte and artificial cerebral spinal fluid, respectively. Vasudevan et al. demonstrated a neural probe with SU-8 waveguide structure for real-time detection of dopamine [70]. Altuna et al. demonstrated an SU-8-based microprobe with planar electrodes and achieved peak-to-peak amplitude ranging up to 400 to 500 µV [71]. Figure 13 shows the SEM images of the fabricated neural probe. Fernández et al. demonstrated a neural device with microfluidic channels and electrode arrays for drug delivery and recording of neural activities all in one platform [72]. It is worth commenting that this work compared the mechanical damage in a rat’s brain produced by the SU-8 device and a standard rigid stereotaxic needle during surgical insertions. Their results led them to conclude that tissue damage by SU-8 is mitigated due to its improved flexibility compared to standard needles.

#### 4.3.3. SU-8-Based Wireless Implantable Devices

As one of the most important physiological parameters in the body, implantable pressure sensors is one the most researched areas. Extensive studies have demonstrated implantable pressure sensors for monitoring intravascular [73], intraocular [9], and intracranial [74] pressure, which may be indicatives of cardiovascular, ocular, or cerebral diseases, respectively. As an implant with minimal invasiveness, these sensors require not only the structural material to be inherently flexible, but also the capability to transmit data wirelessly to ensure chronic implantation.

**Figure 14:**
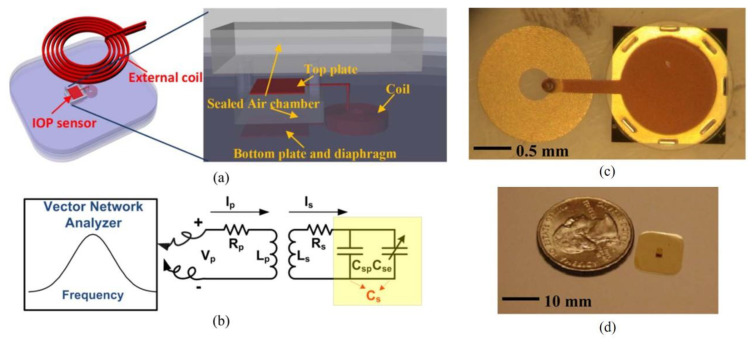
(**a**) Three-dimensional schematic of the wireless IOP sensor system. (**b**) Equivalent circuit of the system. (**c**) Top view of the IOP sensor. (**d**) Size of the IOP sensor compared to a U.S. quarter. © 2012 IEEE. Reprinted from Xue et al. [9] with permission.

Xue et al. demonstrated an SU-8-based battery-free intraocular pressure sensor incorporated with passive inductive coupling principle for wireless sensing [9]. Figure 14 shows the schematic of the wireless pressure sensor and the optical images of the fabricated device. The passive wireless pressure sensor in a size of 1.52 mm × 3.23 mm × 0.2 mm is completely encased in SU-8. The pressure sensor consists of a SU-8 circular diaphragm-based parallel plate capacitor and a circular coil, which forms a *RLC* resonant circuit. When the circular diaphragm is subject to pressure, the deflection of the SU-8 diaphragm causes change of capacitance. This change in capacitance is detectable by their corresponding change in resonant frequency and phase by external radio-frequency (RF) excitation. This battery-free wireless implantable device exhibited a pressure sensing range of 0–60 mmHg with a maximum operation distance of 6 mm between coils in open air.

**Figure 15:**
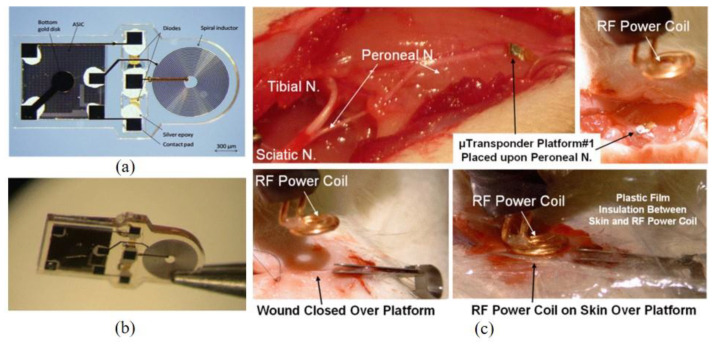
Photomicrographs of the fabricated wireless neurostimulator: (**a**) Conductive epoxy was applied to the socket contact pads, and a couple of Schottky diodes and the ASIC chip were cemented; (**b**) the device was completely sealed by SU-8 and released from the substrate; and (**c**) Acute surgical rat preparation for subcutaneous placement of microstimulator implants to record the cortical response to wireless stimulation of the hind limb. © 2013 IEEE. Reprinted from Cho et al. [75] with permission.

Apart from wireless sensing, inductive coupling technology can also be utilized to transfer power for an application-specific integrated circuit (ASIC) chip. Cho et al. reported a SU-8-based neurostimulator chip consisting of a spiral inductor to wirelessly power the ASIC chip using inductive coupling, Schottky diodes for rectification of RF power, and biphasic platinum-iridium (PtIr) neural stimulation electrodes. All the parts were enclosed in SU-8 [75]. Figure 15 shows the optical images of the fabricated wireless neurostimulator, measuring approximately 3.1 × 1.5 × 0.3 mm. Ex vivo experiments utilized RF frequency operating at 394 MHz coaxially aligned to the spiral inductor and recorded a maximum peak voltage of 6.5 V with 1 W power at 1 mm separation between the external and internal coils. Subsequently, the fabricated device was implanted subcutaneously on the peroneal nerve of a rat. Figure 15 shows the implantation sites as well as in vivo experimental setup in which the external coil was placed over the skin aligned with the implant. At the coupling power of 21 dBm (125 mW) at 394 MHz resonant frequency, stable and robust cortical responses during extended periods of wireless stimulation were recorded using this implanted SU-8-based neurostimulator.

Wireless sensing or power transfer removes the need for a battery or subsequent change of battery through invasive surgeries to ensure long term implantation inside of biological bodies.

## 5. Conclusions

As a versatile polymeric material, SU-8 has been extensively utilized to fabricate innovative MEMS devices, including many unique devices in the biomedical applications. Although the surface biocompatibility of SU-8 might not be completely biocompatible and suffers from toxic leachates, it seems that numerous methods exist to modify the surface of SU-8 to accommodate different needs, such as improved wettability and biocompatibility and the ability to immobilize biomolecules. As a result, SU-8 has widely been utilized in fabricating microstructures that have previously been difficult to achieve for in vitro applications, such as 3D scaffold structures for neuronal cell culturing. Furthermore, SU-8 has allowed biosensors based on immobilization of biomolecules utilizing various detecting principles. Compared to rigid silicon-based devices, functional devices based on SU-8 exhibit lower Young’s modulus and higher yield strength, which make them more suitable to fabricate implantable devices with reduced risks of tissue inflammation and damages. This review paper summarizes the current studies of SU-8 biocompatibility, surface modification techniques, as well as various SU-8-based biomedical devices for in vitro and in vivo applications. It is our view that SU-8 based biomedical devices will gain wider proliferation among the biomedical community in the future, including microfluidics-based lab-on-a-chip and implantable functional device applications.
